# Ceftriaxone compared with penicillin G for the treatment of neurosyphilis: study protocol for a multicenter randomized controlled trial

**DOI:** 10.1186/s13063-022-06769-w

**Published:** 2022-10-01

**Authors:** Fang-Zhi Du, Min-Zhi Wu, Xu Zhang, Rui-Li Zhang, Qian-Qiu Wang

**Affiliations:** 1 Institute of Dermatology, Chinese Academy of Medical Science & Peking Union Medical College, & National Center for STD Control, China Centers for Disease Control and Prevention, No. 12 Jiangwangmiao Street, Xuanwu District, Nanjing, 210042 China; 2 Department of Dermatology, The Fifth People’s Hospital of Suzhou, No. 10 Guangqian Road, Xiangcheng District, Suzhou, 215505 China; 3 Department of Dermatology, The Second Affiliated Hospital of Nanjing Medical University, No. 121 Jiangjiayuan Road, Gulou District, Nanjing, 210011 China

**Keywords:** Neurosyphilis, Treatment, Penicillin G, Ceftriaxone, Multicenter randomized controlled trial

## Abstract

**Background:**

Neurosyphilis may cause irreversible neurological sequelae. First-line treatment consists of penicillin G, with ceftriaxone being an alternative treatment in patients allergic to penicillin. The lack of clinical data comparing the efficacy of these two drugs indicated the need for comparative clinical trials to improve national treatment guidelines in China.

**Methods/design:**

In this multicenter randomized controlled clinical trial, 290 patients newly diagnosed with neurosyphilis will be randomized 1:1 to treatment with aqueous crystalline penicillin G (ACPG) or ceftriaxone. Patients will be treated with standard regimens of ACPG or ceftriaxone according to Chinese National Guidelines and will be followed up for 12 months. All clinical parameters will be assessed at baseline and at follow-up 3, 6, 9, and 12 months later. The primary outcomes will include cerebrospinal fluid (CSF) white blood cell (WBC) count, serological efficacy, and clinical efficacy. The secondary outcomes will include CSF protein concentrations, Mini-Mental State Examination (MMSE) scores, imaging results, recurrence, and time to recovery from neurosyphilis. Adverse events will be monitored and recorded during the trial.

**Discussion:**

This trial will provide clinical data to determine whether ceftriaxone is non inferior to ACPG in treating neurosyphilis and will provide evidence for the improvement of treatment guidelines.

**Trial registration:**

Chinese Clinical Trial Registry ChiCTR2100047164. Registered on 9 June 2021 and updated on 23 November 2021.

## Background

Neurosyphilis is a serious manifestation of syphilis that may cause irreversible damage to the central nervous system (CNS) and even death. The epidemiology of neurosyphilis largely parallels that of syphilis [1, 2]. The reported incidence of syphilis has increased from 30.93 per 100,000 persons in 2014 to 38.37 per 100,000 persons in 2019, an annual increase of 4.41% [3]. The disease burden of neurosyphilis has likely increased to a similar extent, emphasizing the need to eliminate the health hazards caused by neurosyphilis.

Early and adequate treatment of patients with neurosyphilis is the basis for the prevention of neurological sequelae. Penicillin G was first shown in 1940 to be effective in the cure of neurosyphilis [4], and aqueous crystalline penicillin G (ACPG) is still recommended by most guidelines as a first-line treatment for neurosyphilis [5, 6]. Following treatment, however, some patients, especially those with late neurosyphilis, experience persistent symptoms, or their serum or cerebrospinal fluid (CSF) shows no response to nontreponemal tests [7, 8]. Moreover, standard treatment regimens are hard to follow completely, and the long hospital stay and short intervals between ACPG treatments place a considerable burden on patient care [9].

Approximately 10% of patients with neurosyphilis report having an allergy to penicillin [10]. Because ceftriaxone is active against *Treponema pallidum* in vitro, with good blood–brain barrier penetration, ceftriaxone is utilized as an alternative to ACPG to treat neurosyphilis in patients with a penicillin allergy [11]. Ceftriaxone may also be used as outpatient parenteral antimicrobial therapy (OPAT) to reduce the length of hospital stay, providing both economic and quality-of-life benefits to patients with neurosyphilis [12]. A prospective, single-center clinical trial comparing ACPG and ceftriaxone for the treatment of human immunodeficiency virus (HIV)-infected patients with neurosyphilis found no significant differences in serological and clinical efficacy of these two drugs [13]. The conclusions of that study, however, were limited by its small sample size, low number of events, and incomplete data regarding outcomes [14]. Although a recent large, multicenter retrospective study in France found no difference in efficacy between ceftriaxone and ACPG [12], there is a lack of high-quality clinical data to confirm these results [15].

In summary, we designed a non-inferiority randomized controlled trial to compare the efficacy of ceftriaxone with ACPG in the treatment of neurosyphilis. If the findings show that ceftriaxone is non-inferior to ACPG, these will provide evidence for the increased use of ceftriaxone in clinical settings and the improvement of guidelines for the treatment of neurosyphilis. Long-term clinical evaluation may determine whether the efficacy of ceftriaxone is equivalent or superior to that of ACPG.

## Methods

### Study design

**Fig. 1:**
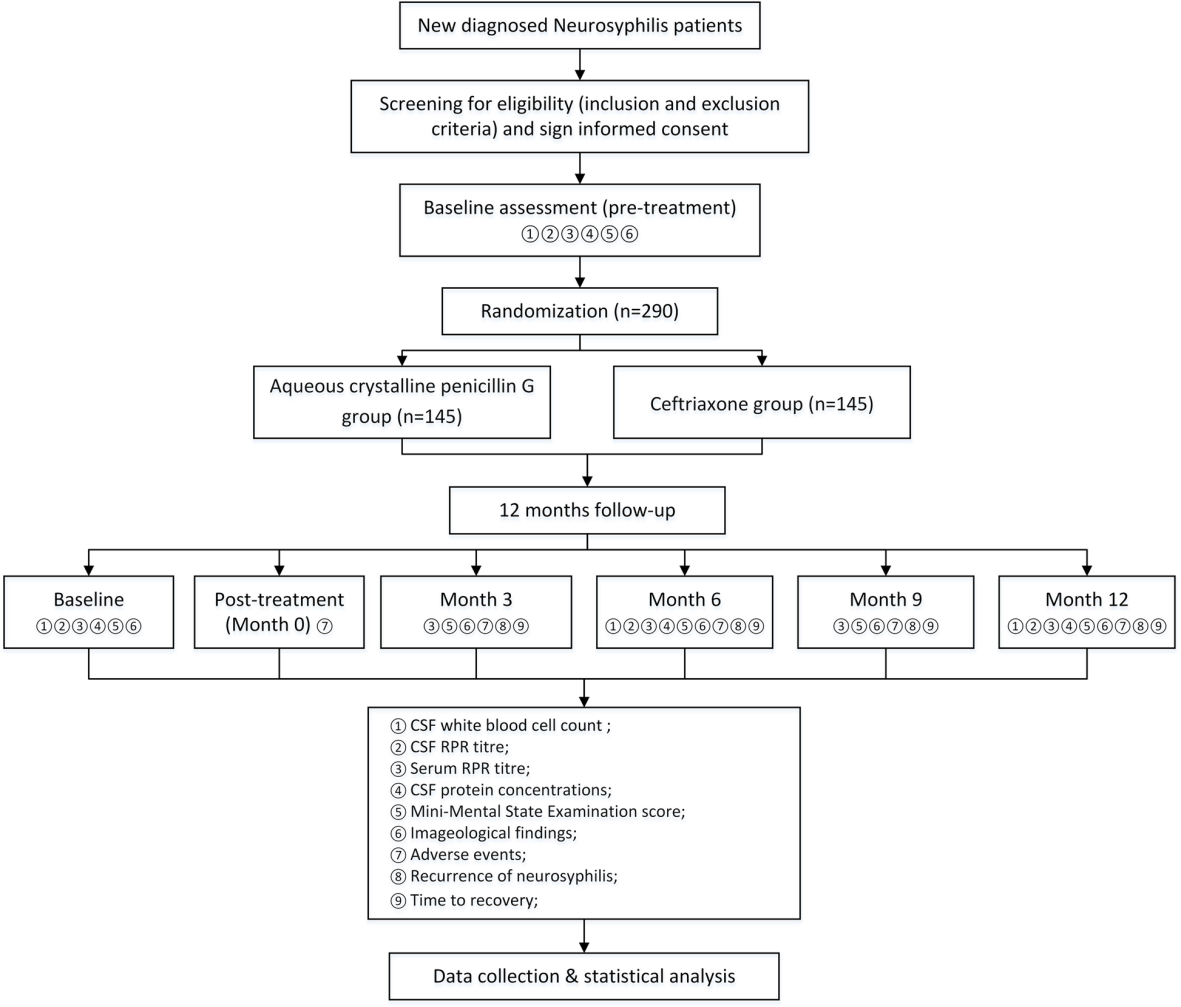
Flow diagram of the study design. Abbreviations: CSF, cerebrospinal fluid; RPR, rapid plasma reagin

**Fig. 2:**
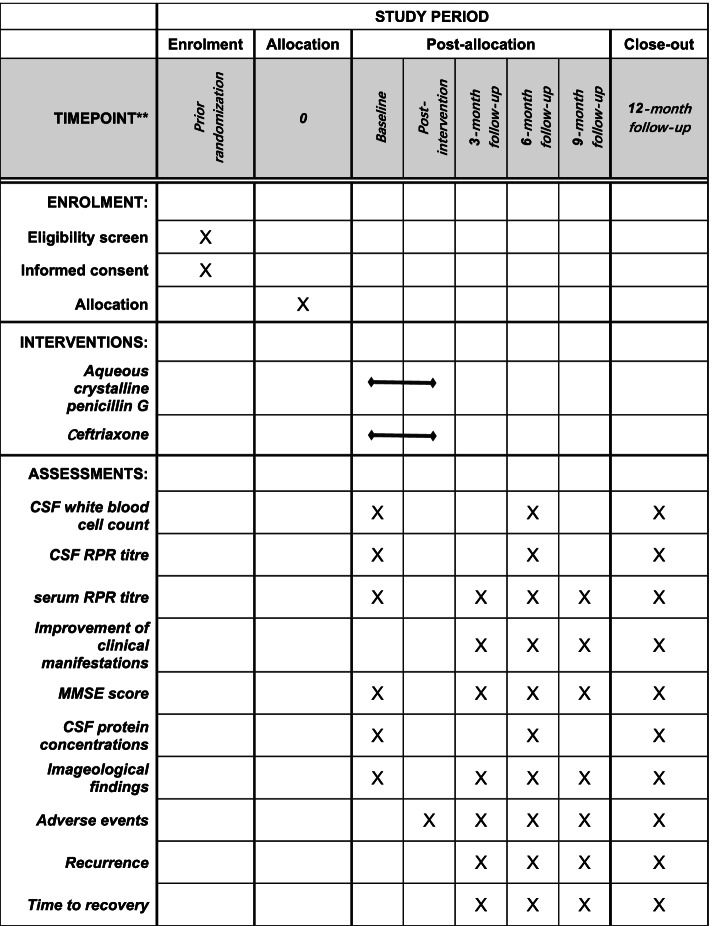
Participant timeline: schedule of enrolment, interventions, and assessments. Abbreviations: CSF, cerebrospinal fluid; RPR, rapid plasma reagin. MMSE, Mini-Mental State Examination

This prospective, multicenter, non-inferiority, randomized, controlled trial was designed to compare the efficacy of ACPG and ceftriaxone in the treatment of patients with neurosyphilis. Patients will be randomized 1:1 to treatment with ACPG or ceftriaxone. Clinical data will be recorded prior to treatment and 3, 6, 9, and 12 months after treatment. The flow diagram of this trial is shown in Fig. 1 and its schedule is shown in Fig. 2. This trial was designed in accordance with the World Health Organization (WHO) Trial Registration Data Set (TRDS) and all relevant items are present in this protocol.

### Participants

This trial was organized by the Institute of Dermatology, Chinese Academy of Medical Science (the head research center) and will be conducted at five medical centers: the Fifth People’s Hospital of Suzhou, the Second Affiliated Hospital of Nanjing Medical University, Chongqing First People’s Hospital, Xi’an North Hospital, and Dalian Dermatology Hospital. Chinese inpatients with newly diagnosed neurosyphilis will be recruited, and all patients will provide written informed consent before treatment and will be randomized into different groups. The trial protocol was reviewed and approved by the Medical Ethics Committee of the Chinese Academy of Medical Sciences Institute of Dermatology and National Center for STD Control in Nanjing (approval number: 2020-KY-022) and has been registered in the Chinese Clinical Trial Registry (ChiCTR2100047164) on 9 June 2021 and updated on 23 November 2021.

#### Inclusion and exclusion criteria

Patients newly diagnosed with neurosyphilis will be enrolled. Criteria for the diagnosis of neurosyphilis include a history of syphilis infection at any stage, with or without CNS manifestations, and laboratory findings that included abnormalities on routine CSF tests, such as elevated white blood cell (WBC) counts, defined as ≥5 cells/mL in the absence or >20 cells/mL in the presence of HIV infection, or protein concentrations of >500 mg/L, and excluding other diseases that may account for these abnormalities [5, 6, 16]. For inclusion, patients had to meet one or more of the following criteria: (i) reactive CSF on Venereal Disease Research Laboratory (VDRL) or fluorescent treponemal antibody absorption (FTA-ABS) tests and (ii) reactive CSF on rapid plasma reagin (RPR)/tolulized red unheated serum tests (TRUST) or Treponema pallidum particle assay (TPPA) tests. Detailed inclusion and exclusion criteria are shown in Table 1. The attending physician of each patient will be responsible for assessing inclusion and exclusion criteria.

**Table 1:** Inclusion and exclusion criteria

**Inclusion criteria**	
1. First-diagnosed neurosyphilis patients (untreated patients referred from other hospitals may also be included)	
2. Age of 18 to 70 years old	
3. No intravenous or intramuscular injection of antibiotics for the treatment of neurosyphilis within the last 3 months (penicillin G, ceftriaxone, etc.)	
4. Patients having voluntarily signed the informed consent form	
**Exclusion criteria**	
1. Allergy to aqueous crystalline penicillin G or ceftriaxone	
2. Exclusion from other central nervous system diseases or conditions that might cause abnormalities in cerebrospinal fluid tests	
3. Pregnancy and lactation	
4. Serious adverse reactions during treatment	
5. Standardized treatment not carried out strictly according to the treatment regimen, or inability to follow up as required	
6. Patients who require the termination of treatment, for which the efficacy and adverse reactions cannot be evaluated, or patients who request withdrawal from the study	
7. Patients with incomplete clinical data that cannot be counted	

### Interventions

#### ACPG group

Patients in the ACPG group will be treated IV with 18–24 million units of ACPG per day, consisting of 3–4 million units every 4 h, for 14 days, followed by intramuscular injections of benzathine penicillin, 2.4 million U per week, for 3 weeks, in accordance with Chinese, American, and European guidelines [5, 6, 16].

#### Ceftriaxone group

Patients in the ceftriaxone group will be treated IV with 2 g ceftriaxone per day for 10–14 days, followed by intramuscular injections of benzathine penicillin, 2.4 million U per week, for 3 weeks, in accordance with previous guidelines [6, 16].

Prior to treatment with ACPG or ceftriaxone, patients will be treated with glucocorticoids to prevent the occurrence of adverse reactions, such as Jarisch–Herxheimer reaction (JHR), as determined by clinicians. Patients with worsening mental disorders or severe adverse events due to treatment were allowed to discontinue treatment. After treatment, patients will be followed up every 3 months for the first year, with clinicians informing patients of return visits by telephone.

### Outcomes

All parameters will be assessed at baseline before treatment (T0) and at follow-up timepoints 3 (T1), 6 (T2), 9 (T3), and 12 (T4) months later.

#### Primary outcomes

##### CSF white blood cell (WBC) count

CSF-WBC count is a sensitive indicator of the curative effect of interventions on neurosyphilis, with Chinese National Guidelines recommending that CSF-WBC count be assessed at 6-month intervals. In this trial, a significant reduction in CSF-WBC count or return to a normal range (<5×10^6^/L in the absence or ≤ 20×10^6^/L in the presence of HIV coinfection) after treatment was defined as effective.

##### CSF syphilitic serological response

In this study, serological cure was defined as a CSF RPR titer that decreased by two dilutions or showed reversion to nonreactivity within 1 year after treatment. CSF RPR titers will be measured at 6 and 12 months after treatment.

##### Serum syphilitic serological response

In this study, clinical cure was defined as a serum RPR titer that decreased by two dilutions or showed reversion to nonreactivity within 1 year after treatment. Serum RPR titers will be measured every 3 months for 1 year after treatment.

##### Improvement of clinical manifestations

Improvements in clinical manifestations are essential in evaluating the efficacy. These improvements will be evaluated among patients with different clinical subtypes of neurosyphilis, such as meningitis syphilis, meningovascular syphilis, general paresis, tabes dorsalis, and ocular syphilis. The presence of the main clinical symptoms of each subtype (Table 2) will be assessed at baseline. Outcomes evaluated during follow-up treatment will include whether each symptom worsened, persisted (no change), recurred (initially improved, but later worsened with no further improvement), improved (clinically relevant improvement without a return to baseline), or recovered (total disappearance of symptoms).

**Table 2:** Clinical manifestations among different subtypes of neurosyphilis

Subtype	Clinical manifestations
**Meningitis syphilis**	Fever
	Headache
	Nausea and vomiting
	Stiff neck
	Meningeal irritation
	Diminution of vision
	Diplopia
	Ptosis
	Facial paralysis
	Hearing loss
	Lower limb weakness
	Paresis, paraplegia
	Gatism
**Meningovascular syphilis**	Hemiplegia
	Aphasia
	Epileptic seizure
	Diffuse pain at the innervation site of the affected nerve
	Defecation disorder
	Hypomyotonia, amyotrophy
**General paresis**	Attention-deficit disorder
	Forgetfulness, poor judgment, and memory
	Cognitive disorder
	Dementia
	Depression
	Personality changes
	Delusion
	Mania
	Amentia
	Epileptic seizure
	Stroke
	Dysarthria
	Hypotonia in the face and limbs
	Involuntary movements of the face, tongue, and hands
**Tabes dorsalis**	Sensory ataxia
	Lightning pain in the lower limbs
	Uroschesis
	Visceral crises
	Lower extremity muscle tone is low
	Charcot joint
	Optic atrophy
	Argyll Robertson pupil
	Tendon reflexes decreased or absent
**Ocular syphilis**	Blepharoptosis
	Limitation of eye movement
	Diplopia
	Diminution of vision
	Blind
	Bulbar conjunctival hyperemia
	Visual field defect
	Metamorphopsia
	Color vision
	Dimmed vision
	Aethomma, floaters

#### Secondary outcomes

##### MMSE score

General paresis is the most frequently reported clinical presentation of neurosyphilis in China [17], with patients having different levels of cognitive impairment. The Mini-Mental State Examination (MMSE), one of the earliest and most widely used brief cognitive assessment tools [18], is utilized to evaluate the severity of dementia among patients with general paresis [19]. In this study, MMSE scores will be determined before treatment and at each follow-up to determine the degree of recovery from cognitive impairment among patients presenting with general paresis at baseline.

##### CSF protein concentrations

Protein concentration in the CSF is a diagnostic indicator of neurosyphilis and will be examined at 6-month intervals. This parameter was defined as a secondary outcome because of its insufficient sensitivity and specificity for the assessment of disease severity [4, 20]. A significant decrease or return to the normal range of CSF protein concentrations (≤ 500 mg/L) will be defined as indicative of effective treatment.

##### Imageological findings

Magnetic resonance imaging (MRI), computed tomography (CT) of the brain, and electroencephalography (EEG) are commonly used as auxiliary examinations for patients with neurosyphilis, with abnormal neuroimaging findings frequently due to CNS inflammation and impairment. Neuroimaging may also show progression in patients who have no neurological symptoms or signs and in patients who receive standardized treatment [21]. In this study, results of patients who undergo imaging examinations before and after treatment will be evaluated. Outcomes will be categorized as no response (no change after treatment), improvement (improved without a return to baseline), and recovery (return to normal).

##### Adverse events

The JHR is the most common transient clinical phenomenon that occurs in patients infected by syphilis who undergo antibiotic treatment. JHR can manifest as fever, chills, rigor, nausea and vomiting, headache, tachycardia, hypotension, hyperventilation, flushing, myalgia, and exacerbation of skin lesions. Although occurring very rarely in patients with late syphilis, the JHR can occur in 75% of patients with general paresis [22]. The present study will compare JHR rates in the ACPG and ceftriaxone groups. Other adverse events due to treatment, such as neurotoxicity and neutropenia, will also be analyzed [23]. All adverse events after treatment and at each follow-up visit will be recorded.

##### Nonresponse to treatment

Nonresponse will be defined as persistent symptoms, a sustained titer of CSF-VDRL/RPR (i.e., the titer did not decrease by two dilutions or showed reversion to nonreactivity), or an elevated CSF-WBC count during the 12-month follow-up.

##### Recurrence of neurosyphilis

Recurrence will be defined as recurrent symptoms or a CSF-VDRL/RPR titer or CSF-WBC count that becomes negative or normal after treatment but subsequently reverts to reactive or abnormal during the 12-month follow-up period.

##### Time to recovery

Time to recovery will be defined as the time to achieve an “overall response” after treatment.

##### Withdrawal

Withdrawal will be defined as any withdrawal for any reason of an individual from this trial during the 12-month follow-up period.

### Sample size

Sample size will be calculated as described [24]. A useful approximate formula is:

$$D={\left(1+R\right)}^2/{\left(1-R\right)}^2\times f\left(\upalpha, \upbeta \right)$$

where *R* is defined as the hazard ratio or risk ratio (RR) and *f*(*α*, *β*) = (*Z*_*β*_ + *Z*_*α*/2_)^2^ equals 7.85 for 80% power, with α = 0.05 and where *Z* is a standardized normal deviation. The RR has been reported to be 1.5 for serological cure (i.e., a two-dilution decrease in CSF-VDRL titer or reversion to nonreactivity) of neurosyphilis treated with ceftriaxone and ACPG [14]. This calculation showed that 196 primary events are required. A recent retrospective study reported that the rates of serological responses in patients treated with ACPG and ceftriaxone were 82% and 88%, respectively [12]. Based on an expected rate of successful treatment of 85%, 230 participants will be recruited. Because rates of loss to follow-up in such clinical trials have not been reported, the loss to follow-up rate in the present trial was set at no more than 20% [25] to ensure the quality of this trial. Thus, a total of 290 patients will be recruited, with 58 at each study center.

### Randomization

Block randomization will be used in the trial. Patients newly diagnosed with neurosyphilis will be randomly assigned 1:1 to the ACPG or ceftriaxone group. The randomization codes will be produced by an independent statistician at the head research center who was unaffiliated with this trial using PROC PLAN through SAS 9.2 software. Each sub-center will be assigned 58 block sequences, and the randomization schedule will be managed by independent investigators not involved in the implementation or statistical analysis of this study.

### Blinding

Because of the obvious differences between modes of administration of ACPG and ceftriaxone, both patients and clinicians will be aware of their group assignment, precluding the possibility of a double-blind study design. However, the outcome assessors and data analysts can be masked (blinded) to the treatment assignment. This prospective randomized open-blinded endpoint (PROBE) study design is frequently used to improve the reliability of analytic results [26].

### Management of data and biological samples

Each research center should assign investigators to collect clinical information from patients, including demographic characteristics (patient name, ID number, telephone number, and other identifying information, which will be kept anonymously), history of sexually transmitted disease (STD) infection and treatment, lifestyle, information acquired at each visit (including clinical manifestations, results of laboratory tests and auxiliary examinations, and diagnosis and treatment information), and the occurrence and management of adverse events. All adverse event data will be systematically collected using standardized coding MedDRA, with the details recorded in a case report form (CRF). At each research center, several researchers will be assigned to call the enrolled patients to remind them of each follow-up visit. Real-time telephone consultations and psychological counseling can be provided after treatment to improve follow-up compliance. All data on each patient will be double entered into an electronic CRF (eCRF). Data from patients who did not complete follow-up and voluntarily withdrew from the study will be retained with other data.

At the end of the trial, the investigators at each branch center will submit the eCRFs of all patients to the head research center. Information on the eCRF for each patient should be complete and signed. All data will be submitted to the Data Monitoring Committee (DMC) of the head research center and uploaded to the Resman Clinical Trial Management Public Platform (http://www.medresman.org.cn/pub/cn/proj/search.aspx) within 6 months after completion of the trial. The results of the trial will be published in a peer-reviewed journal. Documents of all participants will be preserved for at least 10 years after publication, allowing the original data to be accessed if necessary. The clinical data will be used in future studies with the consent of the participants.

The specimens (blood and CSF) used in this trial will be collected by hospital laboratory staff for diagnosis and assessment of the clinical efficacy of treatments for neurosyphilis. After analysis, the samples will be destroyed and will not be kept for future studies.

### Quality control

The head research center is the Institute of Dermatology, Chinese Academy of Medical Science, which has created a standardized operating procedure (SOP) for clinical research to ensure that the treatment protocol is followed throughout the clinical study. Prespecified SOPs for interventions, details about filling out the CRF, result evaluation, and data management will be used to train-related staff members. In addition, quality controllers at each branch center will control the quality of the research tasks performed by each branch center twice per year, such as the implementation of the study protocol, the intervention of the subjects, the completion of the informed consent documents, the filling out of CRF, and the recording and reporting of adverse events. Furthermore, the inspector from the DMC of the head research center will monitor the trial process and the data from each branch research center once per year. If problems are found, the branch research center will be rectified and assessed in strict accordance with the relevant standard operating requirements of this trial. The trial will continue after the branch research center passes the assessment.

### Statistical analysis

All statistical analyses will be performed using SPSS 21.0 statistical software by biostatisticians blinded to group assignment. Missing data for the primary outcome will be imputed using the multiple imputation method. Serum and CSF RPR titers will be analyzed after log_2_ conversion. Normally distributed continuous variables in the two groups will be compared by *t* tests, and categorical variables will be compared by the *χ*^2^ test or *Fisher’s *exact test, as appropriate. Nonparametric variables will be compared by rank sum tests. The correlation between serum and CSF RPR titers will be analyzed using Spearman’s rank correlation tests. Based on the assumption that there are no obvious correlations between primary outcomes and that it will be impossible to determine which outcome has greater weight, the weights of these primary outcomes will be evaluated using the “Experts Scores” algorithm, with “overall response” to treatment evaluated according to the total efficacy scores from these primary outcomes. The results of each primary outcome will also be evaluated separately. The non-inferiority margin will be set at 10 percentage points for the absolute difference between the two groups. If the standard for non-inferiority is reached, all primary and secondary outcomes will be tested for superiority (*P*<0.05). Non-inferiority will be considered reached if conclusions drawn from the intention-to-treat (ITT) population, consisting of all patients who met the inclusion criteria, were randomized, and started treatment, and the per-protocol population, consisting of all patients with good compliance and who followed the protocol to completion of the treatment regimen, are consistent. Univariate analyses will be performed to identify potential factors associated with the treatment outcome, with variables having a *P* value ≤0.10 in univariate analyses included in a multivariate logistic regression model. Odds ratios (ORs), adjusted ORs (aORs), and their 95% confidence intervals (CIs) will be calculated. A two-sided *P value* <0.05 will be considered statistically significant. Because the efficacy and safety of ceftriaxone and ACPG are clear and both are recommended by Chinese National Guidelines, this trial will not include interim analyses.

## Discussion

Penicillin G is regarded as the first-line drug for the treatment of neurosyphilis based on the pharmacokinetics of available drugs, their effect on *T. pallidum* in vitro, laboratory considerations, biological plausibility, expert opinions, case studies, and clinical experience [5]. Ceftriaxone is an alternative to penicillin G for the treatment of patients with penicillin allergy. Clinically, the short time interval between ACPG treatments and the long duration of hospital stay increase the burden of patient care, such that many patients prefer the ceftriaxone regimen. However, there is a lack of clinical data comparing the efficacy of these two drugs. To our knowledge, this trial will be the first multicenter clinical trial comparing ACPG with ceftriaxone in a large number of patients in China with neurosyphilis. Findings of this trial may provide evidence to improve treatment guidelines.

This trial will meet high methodological standards, such as strict inclusion and exclusion criteria, adequate randomization and concealment of allocation, a PROBE design, the inclusion of independent statisticians, and multilevel quality control. These factors increase the credibility of the clinical trial results. Second, comprehensive outcomes will be evaluated to compare the efficacy of ACPG and ceftriaxone. Previous clinical trials have evaluated the efficacy of treatment for neurosyphilis based on rates of serological cure, clinical cure, and normalization of CSF-WBC counts and on protein concentrations [17, 27–30]. Several studies have also evaluated the use of neuroimaging examinations to determine the efficacy of neurosyphilis treatments [21, 31]. The present trial will also evaluate clinical improvements (including MMSE scores), adverse events, time to recovery, and recurrence of neurosyphilis. Evaluation of improvements in clinical manifestations will include improvements of the most common clinical manifestations of each clinical subtype of neurosyphilis during follow-up. These parameters have not yet been evaluated in previous studies and may provide strong evidence to determine the efficacy of treatment. Adverse events will be assessed to determine the safety of ceftriaxone. Disease recurrence, time to recovery, and 1-year follow-up results in patients with neurosyphilis may provide evidence for evaluating the long-term efficacy of the two drugs. In addition, because neurosyphilis patients are frequently lost to follow-up, the sample size calculated using a standardized formula has been increased to improve the reliability of outcomes. Finally, analysis of clinical factors, such as smoking, alcoholism, stage of disease (early or late neurosyphilis), asymptomatic or symptomatic status, baseline serum and CSF RPR titer [32], and CSF-WBC counts and protein concentration, may enable the identification of the factors related to treatment efficacy and obtain evidence for providing a more personalized and accurate treatment regimen.

This trial, however, will also have several important limitations. First, the US and European treatment guidelines do not include the optimal follow-up times for evaluating treatment of neurosyphilis [5, 16]. Moreover, the Chinese National Guidelines state that the follow-up time should be more than 3 years [6]. Because the number of patients lost to follow-up will increase over time, the present study will include a follow-up time of only 1 year, consistent with previous studies [14, 29]. Second, due to the differences between the two treatment regimens, a double-blind trial design cannot be implemented. Third, although improvements in clinical manifestations will be comprehensively evaluated, these manifestations cannot be determined quantitatively. Moreover, the efficacy of ACPG and ceftriaxone will not be compared with the efficacy of other drugs, such as doxycycline and tetracycline. Consequently, additional clinical trials of these other drugs will be needed in the future.

In summary, this trial will compare the efficacy of ceftriaxone and ACPG in the treatment of patients with neurosyphilis, especially focusing on the clinical effectiveness and safety of ceftriaxone. Clinical data obtained from this trial may provide evidence for improvements in treatment guidelines.

## Trial status

Chinese Clinical Trial Registry ChiCTR2100047164, version 2. Registered on 9 June 2021 and last updated on 23 November 2021. Participant recruitment began on 1 December 2021 and is expected to continue until 2024.

## Data Availability

All data will be uploaded to the Resman Clinical Trial Management Public Platform (http://www.medresman.org.cn/pub/cn/proj/search.aspx) within 6 months after completion of the trial and are available upon request from the authors.

## Abbreviations

CNS: Central nervous system
ACPG: Aqueous crystalline penicillin G
CSF: Cerebrospinal fluid
HIV: Human immunodeficiency virus
VDRL: Venereal Disease Research Laboratory
FTA-ABS: Fluorescent treponemal antibody absorption
RPR: Rapid plasma reagin
TRUST: Tolulized red unheated serum test
TPPA: Treponema pallidum particle assay
JHR: Jarisch–Herxheimer reaction
WBC: White blood cell count
MMSE: Mini-Mental State Examination
MRI: Magnetic resonance imaging
CT: Computed tomography
EEG: Electroencephalography
RR: Risk ratio
CRF: Case report form
eCRF: Electronic CRF
DMC: Data Monitoring Committee
SOP: Standardized operating procedure
ORs: Odds ratios
aORs: Adjusted ORs
CIs: Confidence intervals
